# Cytometric profiling in various clinical forms of multiple sclerosis with respect to CD21^+^, CD32^+^, and CD35^+^ B and T cells

**DOI:** 10.1186/2047-9158-2-14

**Published:** 2013-07-02

**Authors:** Ali Zandieh, Maryam Izad, Mohammad Fakhri, Hamed Amirifard, Zahra Khazaeipour, Mohammad Hosein Harirchian

**Affiliations:** 1 Department of Neurology, Imam Khomeini Hospital Complex, Tehran University of Medical Sciences, 14197, Tehran, Iran; 2 Iranian Center of Neurological Research, Tehran University of Medical Sciences, Tehran, Iran; 3 Department of Immunology, School of Medicine, Tehran University of Medical Sciences, Tehran, Iran; 4 Brain and Spinal Cord Injury Research Center, Tehran University of Medical Sciences, Tehran, Iran

**Keywords:** CD21, CD32, CD35, B Cells, T Cells, Multiple Sclerosis

## Abstract

**Background:**

We aimed to evaluate the frequency of various types of B and T cells expressing CD21, CD32, and CD35 in multiple sclerosis (MS) clinical courses.

**Methods:**

Peripheral blood mononuclear cell from 30 MS patients (17 relapsing remitting [RRMS], six secondary progressive [SPMS], and seven primary progressive MS [PPMS]) and 18 healthy subjects were analyzed. All patients were in acute attack. Healthy controls were matched for age and gender ratio. The frequencies of various subsets of B and T cells were determined using flow cytometry.

**Results:**

The frequency of CD4^+^T cells was lower in MS patients compared to control subjects (41.14 ± 9.45% vs. 46.88 ± 6.98%, respectively, *P* < 0.05). The CD32^+^ fraction of CD4^+^T cells and the CD21^+^ fraction of CD8^+^T cells were higher in MS patients (2.85 ± 3.72% vs. 1.06 ± 0.62% for CD32^+^CD4^+^T cells, 2.71 ± 1.86% vs. 1.16 ± 0.99% for CD21^+^CD8^+^T cells in MS patients and control subjects, respectively, *P* < 0.05). After dividing subjects by type of MS course, higher values of these two T cell subsets were found in SPMS patients compared to control subjects (*P* < 0.05). Further, RRMS patients had lower levels of CD32^+^CD4^+^T cells than SPMS patients and also they had lower levels of CD32^+^CD8^+^T cells than PPMS patients (*P* < 0.05). However, neither the expression of CD35 on T cells nor the various B cell subsets were statistically different between the compared groups.

**Conclusions:**

Our findings demonstrate that T cell subsets expressing CD21 and CD32 may differ with respect to the presence or clinical forms of MS disease. By contrast, CD35^+^T cells and different subsets of B cells are not altered in various MS clinical courses.

## Background

Multiple Sclerosis (MS) is a chronic autoimmune demyelinating disease, which imposes huge burden on quality of life and socioeconomic status of patients [1-3]. MS manifests itself in various clinical forms (e.g. relapsing remitting MS [RRMS], secondary progressive MS [SPMS], and primary progressive MS [PPMS]) and its course in each individual is varied and unpredictable [1,4].

Although the exact pathogenesis of the disease has not yet been completely elucidated, some evidence indicates inflammation and immune system as the primary causes of destruction of the myelin sheath and axonal loss [5-7]. Review of previous studies shows that various clusters of differentiation antigens (CDs, i.e. antigens present on leukocytes with particular lineage and differentiation stage) may be involved in the pathogenesis of MS. For instance, CD25 inhibition is suggested as a possible treatment for RRMS and some other autoimmune diseases [8]. Or else, CD161 expression on CD8^+^ T cells may contribute to the MS pathogenesis [9]. In this respect, other subsets of B and T cells (e.g. CD29^+^, CD44^+^, and CD45RA^+^) have been also evaluated in MS patients [10-12].

Immune complexes, as an important part of immune system, are formed due to the interaction of antigens and antibodies [13]. These complexes can influence immune system via CD21 and CD35 (also alternatively called complement receptor II and I, respectively) and CD32 (i.e. Fcγ receptor II) [13,14]. It has been suggested that change in the expression or function of these receptors may contribute to some autoimmune disorders (e.g. lupus, rheumatoid arthritis and sjogren’s syndrome) [15-19]. However, no previous study has evaluated fractions of cells expressing each of these markers among B cells and CD4^+^ or CD8^+^ T cells in MS patients. Further, for the first time we stratified MS patients according to the disease course and compared various types of B and T cells between them.

## Methods

### Subjects

Thirty consecutive patients diagnosed with MS according to McDonald criteria [20], at the MS clinic of Imam Khomeini hospital (a Tehran University affiliated medical center) were included in this study from January 2010 to January 2012. Only active RRMS patients who experienced MS exacerbation within the preceding two weeks were included. PPMS and SPMS patients had active progression. Exacerbation and progression were approved by the treating experienced neurologists in accordance with the criteria of McDonald [20]. Patients who received interferon and/or corticosteroids within past three months, or those with previous history of hypersensitivity or history of recently acquired infectious diseases (e.g. respiratory and gastrointestinal infections) were excluded. For each patient the type of MS was determined according to the Lublin–Rheingold classification [21]. Disability was assessed by an experienced neurologist using Expanded Disability Status Scale (EDSS) [22]. Using propensity algorithm 18 subjects were selected as controls from those who were visited at the offices of Blood Transfusion Organization of Tehran for blood donation. The control group was matched for age and sex ratio with MS patients. These healthy controls were screened for neurological problem, recently acquired infectious diseases, or familial history of MS. All subjects gave written informed consent before study commencement. The study was performed in agreement with Helsinki declaration and was conducted in accordance with considerations, recommended by local ethics review committee of Tehran University of Medical Sciences.

### Flow cytometry and cell sorting

For flow cytometry, aliquots of 1 × 10^6^ peripheral blood mononuclear cells were isolated from fresh blood samples and stained with following anti-human antibodies: anti-CD4-PE-Cy5, anti-CD8-PE-Cy5, anti-CD19-PE-Cy5, anti-CD21-PE, anti-CD32-FITC, anti-CD35-PE, as well as appropriate isotype control antibodies (eBioscience, San Diego, CA). In brief, recommended quantity of each primary antibody was combined with an appropriate volume of flow cytometry staining buffer, so that the antibody mixture reached the volume of 50 μL. Then, this mixture was added to wells containing 50 μL of cell suspension. Flow cytometry was performed on samples using a FACSCalibur flow cytometer (BD Biosciences, San Jose, CA). The presence of surface antigens was expressed as the proportion of positive stained cells.

### Statistical analysis

Data were analyzed in SPSS software (version 18.0; SPSS Inc., Chicago, USA). Variables were compared using Student’s t-test, or one-way Anova, followed by Scheffe post-hoc comparisons. Results with *P* < 0.05 were considered statistically significant.

## Results

The mean age ± standard deviation (SD) of MS patients and control subjects were 37.53 ± 8.87 and 36.72 ± 7.08 years, respectively (*P* > 0.05). The proportions of men in both groups were 33.3% equally. EDSS of MS patients was estimated to be 2.98 ± 1.64 unites. According to the Lublin–Rheingold classification, 17 (56.7%), 6 (20.0%) and 7 (23.3%) MS patients were diagnosed to have RRMS, SPMS and PPMS, respectively (Table 1). Age, EDSS and disease duration differed across various subtypes of MS disease (*P* < 0.05). Post-hoc analysis revealed that patients with RRMS were younger and had lower EDSS scores compared to those with SPMS (*P* < 0.05). Further, age and duration of disease of RRMS patients were lower than those who had PPMS (*P* < 0.05).

**Table 1:** Principal characteristics of the MS patients stratified by disease subtype

	**RRMS (*n*** **=** ***17*)**	**SPMS (*n*** **=** ***6*)**	**PPMS (*n = 7*)**
Age (years)	32.3 ± 7.10	41.7 ± 5.28 ^a^	46.6 ± 5.91 ^a, b^
EDSS (unites)	2.16 ± 1.48	4.50 ± 1.38 ^a^	3.64 ± 0.94 ^b^
Disease duration (years)	3.12 ± 3.46	7.83 ± 4.31	8.86 ± 6.36 ^a, b^

Data are presented as mean ± standard deviation (SD).

^a^ P < 0.05 compared to RRMS.

^b^ P < 0.05 for the comparison between three groups.

Table 2 shows fractions of studied cells in the whole blood stained mononuclear cells. On the contrary to CD8^+^ T cells and CD19^+^ B cells which were not statistically different between MS patients and controls, CD4^+^ T cells had a lower level in MS patients (Table 2). In this regard, there were not significant differences in patients with various clinical forms of MS.

**Table 2:** The fractions of various subsets of cells among whole stained mononuclear cells in MS patients and control subjects

	**Controls (*n = 18*)**	**MS**			
		**Overall (*n = 30*)**	**RRMS (*n = 17*)**	**SPMS (*n = 6*)**	**PPMS (*n = 7*)**
CD4^+^ T cells	46.88 ± 6.98	41.14 ± 9.45 ^a^	41.76 ± 9.08	42.75 ± 4.45	37.85 ± 14.04
CD8^+^ T cells	26.20 ± 3.02	29.13 ± 7.42	26.35 ± 7.48	33.60 ± 4.69	31.27 ± 7.34
CD19^+^ B cells	7.62 ± 2.33	8.26 ± 3.35	8.81 ± 3.38	6.77 ± 4.22	8.38 ± 2.20

Data are presented as mean percentage ± SD.

^a^*P* < 0.05 compared to control subjects.

**Figure 1:**
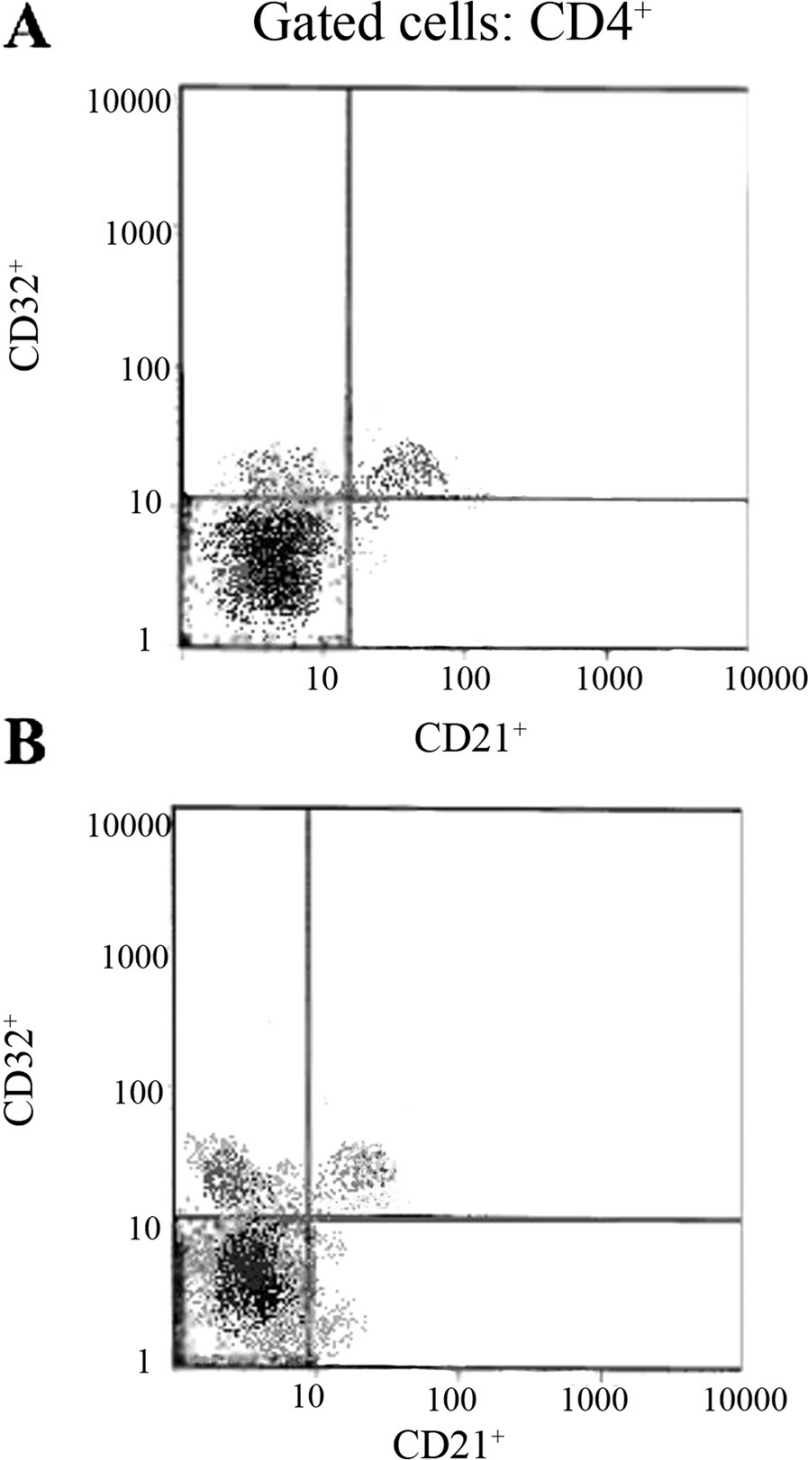
**Immunofluorescence analysis of CD4**^**+ **^**gated lymphocytes in Control subjects (A) and MS patients (B).** As it is evident in the left upper quadrants of both dot plots, the percentage of CD32^+^ of the total population of CD4^+^ cells is higher in MS patients.

**Figure 2:**
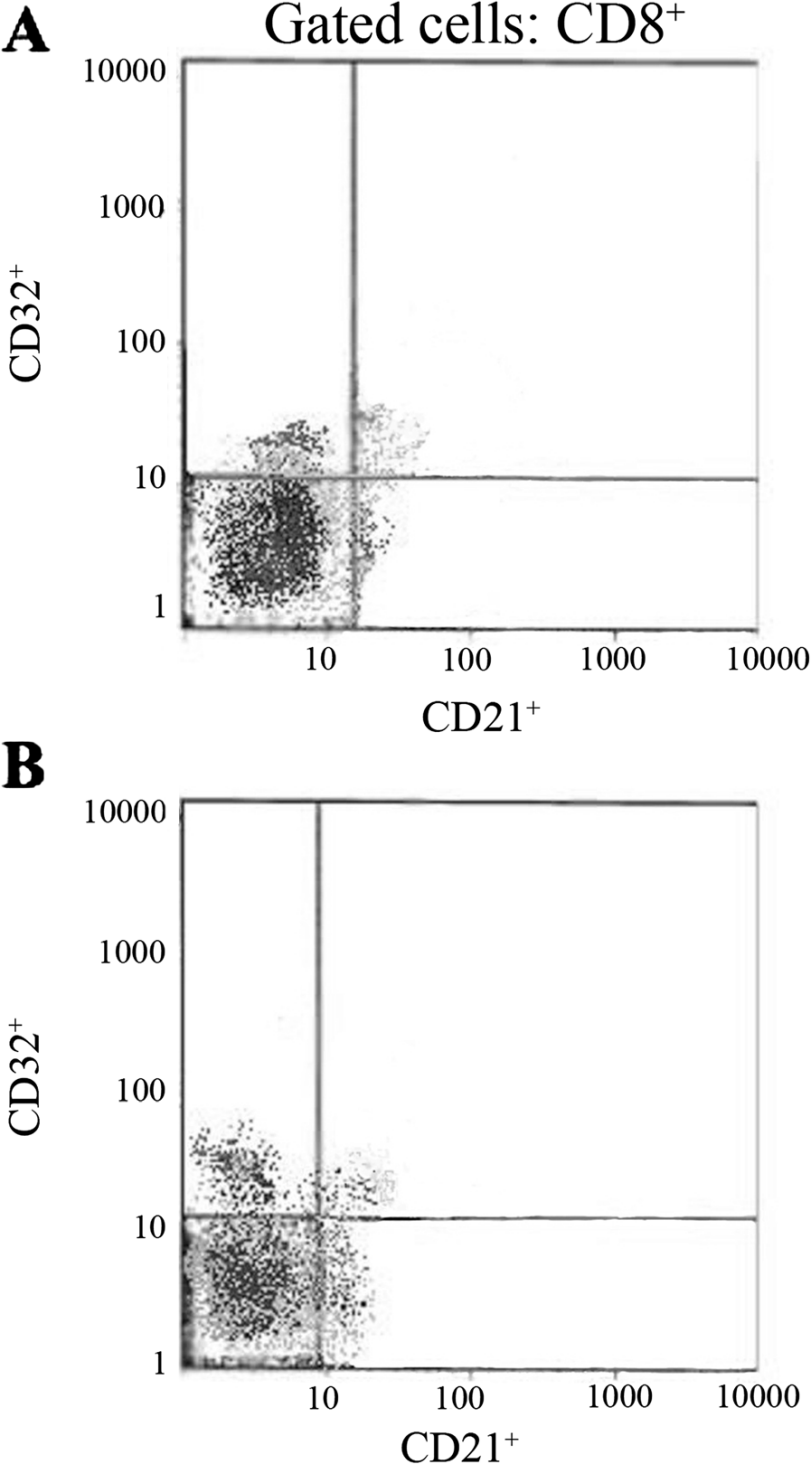
**Immunofluorescence analysis of CD8**^**+ **^**gated lymphocytes in Control subjects (A) and MS patients (B).** As it is evident in the right lower quadrants of both dot plots, the percentage of CD21^+^ of the total population of CD8^+^ cells is higher in MS patients.

Fractions of each subtype of cells among CD4^+^ or CD8^+^ T cells or CD19^+^ B cells were measured (Table 3). We found that CD21, CD32 and CD35 markers are more likely to be detected on CD19^+^ B cells than on CD4^+^ or CD8^+^ T cells. CD21CD32 double staining of peripheral lymphocytes in control subjects and MS patients are shown in Figures 1 and Figure 2. CD32^+^CD4^+^ and CD21^+^CD8^+^ T cells were increased in MS patients (*P* < 0.05). Other cell populations were not statistically different between MS patients and control subjects (*P* > 0.05). The fraction of CD32^+^ cells among CD4^+^ T cells was increased in SPMS compared to RRMS and control subjects (*P* < 0.05). However, this difference did not reach significant levels between SPMS and PPMS patients. Patients with SPMS disease had higher proportion of CD21^+^CD8^+^ T cells than control subjects (*P* < 0.05). The proportion of CD8^+^ T cells which also expressed CD32 was higher in PPMS compared to RRMS (*P* < 0.05). The differences in the levels of CD32^+^CD8^+^ T cells between PPMS and control subjects showed a trend but didn’t reach significance levels (*P* = 0.051). Finally, the expression of CD35 did not differ significantly between various subtypes of MS disease.

**Table 3:** **The fractions of various subsets of cells among the CD4**^**+ **^**or CD8**^**+ **^**T cells, or CD19**^**+ **^**B cells**

	**Controls (*n = 18*)**	**MS**			
		**Overall (*n = 30*)**	**RRMS (*n = 17*)**	**SPMS (*n = 6*)**	**PPMS (*n = 7*)**
CD21^+^ in CD4^+^ T cells	0.41 ± 0.34	0.77 ± 0.76	0.78 ± 0.76	0.79 ± 1.09	0.70 ± 0.42
CD32^+^ in CD4^+^ T cells	1.06 ± 0.62	2.85 ± 3.72 ^a^	1.68 ± 1.05	5.97 ± 7.40 ^a, b^	2.86 ± 1.65
CD35^+^ in CD4^+^ T cells	5.34 ± 4.74	8.73 ± 8.85	8.33 ± 5.33	5.52 ± 2.15	12.36 ± 16.01
CD21^+^ in CD8^+^ T cells	1.16 ± 0.99	2.71 ± 1.86 ^a^	2.07 ± 0.66	4.08 ± 3.33 ^a^	2.92 ± 1.35
CD32^+^ in CD8^+^ T cells	3.05 ± 2.52	3.22 ± 3.03	1.88 ± 1.82	2.98 ± 1.95	6.29 ± 3.87 ^b, c^
CD35^+^ in CD8^+^ T cells	4.43 ± 1.95	5.43 ± 3.38	5.65 ± 4.48	5.26 ± 1.80	5.23 ± 2.48
CD21^+^ in CD19^+^ B cells	84.76 ± 9.66	87.51 ± 6.67	88.13 ± 6.69	86.07 ± 8.20	87.05 ± 5.37
CD32^+^ in CD19^+^ B cells	70.02 ± 13.77	66.46 ± 15.42	63.39 ± 16.65	65.90 ± 14.42	74.41 ± 11.67
CD35^+^ in CD19^+^ B cells	94.43 ± 4.90	92.63 ± 5.06	93.61 ± 4.58	92.27 ± 1.97	90.57 ± 7.55

Data are presented as mean percentage ± SD.

^a^*P* < 0.05 compared to control subjects.

^b^*P* < 0.05 compared to RRMS.

^c^*P* = 0.051 compared to control subjects.

## Discussion

In the present study we evaluated various CD21-, CD32- and CD35-related subsets of B and T cells in MS patients. Our data demonstrated that the level of CD4^+^ T cells was lower in MS patients. The fractions of CD32^+^ and CD21^+^, respectively among CD4^+^ and CD8^+^ T cells were increased in MS patients compared to control subjects. SPMS patients had higher frequency of CD32^+^CD4^+^ T cells in comparison to RRMS and control subjects. The level of CD21^+^CD8^+^ was also increased in patients with SPMS. Likewise, PPMS patients had higher level of CD32^+^ cells among CD8^+^ T cells compared to RRMS. Nevertheless, none of the comparisons made between various subsets of B cells reached significant levels.

As we stated earlier, CD21 is related to some autoimmune disorders. For instance, the level of CD21 in serum of patients with lupus, rheumatoid arthritis, Sjogren’s syndrome, and systemic sclerosis is decreased [19,23,24]. In addition, there is some evidence regarding the role of CD21 in the pathogenesis of MS. For instance, CD21 serves as a receptor for Epstein-Barr virus, which *per se* is suggested to be related to the MS [25,26]. Toepfner *et al*. showed that CD21 level is altered in MS patients [27]. Further, change in percentage value of CD21^+^ cells is suggested as a marker of immune system activation in MS patients [10]. Comparing fractions of cells expressing CD21 among B cells, CD4^+^, or CD8^+^ T cells, we found that merely CD21^+^CD8^+^ cells are elevated in MS patients. Thus, it may be plausible to suggest CD8^+^ T cells as chief cells that associate CD21 with MS disease. Considering the influence of age and interferon-β therapy on CD21 [23,27,28], one possible strength of the current study is that we controlled the effects of these two potentially confounding variables.

Our results demonstrate that the level of CD32^+^ cells may differ between MS patients and control subjects, and also between subgroups of patients. CD32 is highly expressed on microglia cells in MS lesions [29] and it may also be involved in remyelination of these lesions [30]. Besides conflicting data, CD32 polymorphism may also be associated with MS [31,32]. In general agreement with aforementioned findings, our results suggest a role for CD32 in the pathogenesis of MS. We found that the expression of CD32 on both CD4^+^ and CD8^+^ T cells is altered in progressive courses of MS in comparison to RRMS. By contrast, there was not significant difference in the level of B cells with respect to CD32. This point is supported by findings of Cambella *et al*. who revealed that expression of CD32 among B cells is not influenced by MS disease [33].

In a similar way to CD21 and CD32, CD35 is also related to some autoimmune diseases [34,35]. It had been shown that soluble recombinant CD35 improves inflammation, demyelination, and severity of clinical disease in a rat model of MS disease [36]. However, apart from the possible therapeutic relevance of CD35, Vedeler *et al*. found no association between the cerebrospinal fluid and serum levels of this marker with activity or clinical forms of MS disease [37]. Similarly, after comparing the frequency of CD35^+^ cells in our study, we found that various types of CD35^+^ cells are not statistically different between controls and MS. Thus, in comparison to CD21 and CD32, considering a pathophysiologic role for CD35 in development of MS is more doubtful.

The main limitation of the current study is the low number of enrolled SPMS and PPMS patients. One reason that holds us back from enrolling higher number of patients was the exact inclusion criteria of the current study. Considering the influence of corticosteroid- and interferon-therapy on immune system we decided not to include those patients who recently have received these treatments. Thus, many patients especially with primary-progressive and secondary-progressive clinical forms were disqualified from entering our study.

## Conclusions

In summary, we compared CD21, 32 and 35 markers on B and T cell population in MS patients and found that the fraction of CD32^+^ cells among CD4^+^ T cells and the fraction of CD21^+^ cell among CD8^+^ T cells are higher in MS patients than control subjects. RRMS patients have lower levels of CD32^+^ cells among CD4^+^ and CD8^+^ T cells compared to SPMS and PPMS patients, respectively. However, MS patients did not show a difference in the frequency of CD35^+^ cells. The current study for the first time evaluates various subsets of B and T cells expressing CD21, CD32 and CD35 in patients with different clinical courses of MS and may help in developing a disease biomarker and diagnostic tool for MS and its different clinical forms. Further, our findings along with other studies in this line may help to elucidate the underlying pathophysiologic pathway(s) which link clinical courses of MS and immune function. Further studies should also be designed to evaluate how change in the number of various B and T cell subtypes might influence the appearance of soluble forms of these markers in serum.

## Abbreviations

EDSS: Expanded disability status scale; MS: Multiple sclerosis; PPMS: Primary progressive multiple sclerosis; RRMS: Relapsing remitting multiple sclerosis; SPMS: Secondary progressive multiple sclerosis.

## Competing interests

The authors declare that they have no conflict of interests.

## Authors’ contributions

AZ designed the study, participated in the coordination and acquisition of data, wrote the manuscript and performed the statistical analysis. MI conceived the study, participated in the coordination and acquisition of data and supervised the study process. MF helped to draft the manuscript and participated in the coordination and acquisition of data. HA helped to draft the manuscript and interpret the data and participated in the acquisition of data. ZK helped to perform the statistical analysis. MHH designed and supervised the study process. All authors read and approved the final manuscript.
